# Evaluating the causal effects between Grave’s disease and diabetes mellitus: a bidirectional Mendelian randomization study

**DOI:** 10.3389/fendo.2024.1420499

**Published:** 2024-11-06

**Authors:** Yuhan Zhang, Liuxiang Fu

**Affiliations:** ^1^ Emergency Department, The Second Affiliated Hospital, Jiangxi Medical College, Nanchang University, No.1 Minde Road, Nanchang, China; ^2^ General Surgery Center, Department of Thyroid Surgery, The 1^st^ Hospital of Jilin University, Chang Chun, China; ^3^ Department of General Surgery, Panzhihua Central Hospital, Panzhihua, China

**Keywords:** Graves’ disease, Mendelian randomization, type 1 diabetes, type 2 diabetes, autoimmune disease

## Abstract

**Background:**

Graves’ disease (GD) is an autoimmune disease associated with an increased incidence of other autoimmune diseases. To investigate the causality between GD and Diabetes mellitus (DM), we designed bidirectional two-sample Mendelian randomization (MR) and multivariable MR (MVMR) studies.

**Methods:**

Single-nucleotide polymorphisms (SNPs) associated with GD, thyroid peroxidase (TPO), thyroglobulin (Tg), thyroid-stimulating hormone (TSH), type 1 diabetes (T1D), and type 2 diabetes (T2D) were obtained from the IEU Open GWAS and FinnGen biobank databases. For the forward MR study, we used GD (sample size = 458,620) as the exposure and T1D (sample size = 520,580) and T2D (sample size = 211,766) as the outcomes. Next, high risk of T1D and T2D were used as exposure variables, and GD was used as the outcome variable for the reverse MR analysis. Finally, MVMR analysis was conducted to investigate the probable relationship between DM and indicators for thyroid function like TPO, Tg, and TSH. The inverse variance weighting (IVW) was used as the main method. Finally, the heterogeneity and sensitivity were assessed.

**Results:**

There were 27, 88, and 55 SNPs associated with GD, T1D, and T2D, respectively. A significant causal connection between higher genetic liability of GD and the risk of T2D (OR [95% CI] = 1.059 [1.025–1.095], P = 5.53e-04) was found in the forward MR analysis. Comparatively, the significant causal relationship between higher genetic liability of GD and the risk of T1D was not demonstrated (OR [95% CI] = 0.998[0.927,1.074], P=0.949). However, reverse MR suggested that there was a genetic susceptibility to T1D that increased the likelihood of developing GD (OR [95% CI] = 1.173[1.117,1.231], P = 1.913e-10), while T2D did not (OR [95% CI] = 0.963 [0.870–1.066], *P* = 0.468). Furthermore, there was inadequate evidence to suggest that abnormal TSH, TPO, and Tg levels increase the risk of incident T1D or T2D in individuals with GD. MVMR revealed no causal relationship among Tg, TSH, TPO, T1D, or T2D.

**Conclusion:**

There was no increased risk of T1D with an increase in genetic susceptibility to GD, although higher genetic susceptibility to T1D has been shown to be associated with increased risk of developing GD. A unidirectional causal relationship between the genetic liability for GD and increased risk of T2D was observed using MR analyses. MVMR analysis showed no statistically relevant causality between the genetic liability for TSH, TPO, or Tg and the risk of either T1D or T2D.

## 1 Introduction

Diabetes mellitus (DM), a metabolic disorder characterized by high blood glucose levels, is one of the fastest-growing global health problems of the 21st century. Globally, 537 million people were estimated to have diabetes by 2021. By 2030, this figure is likely to rise to 643 million, and by 2045, it is predicted to reach 783 million (1). Associated complications of DM affect multiple organs and include cardiovascular disease, diabetic nephropathy, retina-related eye diseases, neuropathy, and diabetic foot ulcers (2, 3). There are three broad forms of DM: type 1 (T1D), type 2 (T2D), and gestational diabetes. T1D is an autoimmune illness mostly caused by the immune system attacking the β-cells in the pancreas that produce insulin (4–6). T1D mainly occurs in pediatric patients; however, some patients with latent autoimmune diabetes are diagnosed during adulthood (7). T2D is mostly caused by insulin resistance (8) and is distinguished by the absence of pathological alterations in the pancreas and insensitivity of other body parts and tissues to insulin. Obesity is believed to be the primary cause of T2D (9). However, some studies have demonstrated that immunology also contributes to its pathophysiology (10–13). Owing to its high incidence and numerous complications, DM has a considerable influence on the quality of life of patients and is a heavy strain on society. Investigating the risk factors for DM development and implementing early preventive interventions are crucial.

Diffuse toxic goiter, also known as Graves’ disease (GD), is an autoimmune condition that affects the thyroid glands. Hyperthyroidism is mainly caused by GD (14). Many patients with GD exhibit abnormal levels of indicators of thyroid function, thyroid-stimulating hormone (TSH), thyroid peroxidase (TPO), and thyroglobulin (Tg). Patients with GD typically present with a range of hypermetabolic symptoms due to elevated thyroid hormone levels, including hyperthermia, hyperhidrosis, irritability, and cardiac arrhythmias, such as atrial fibrillation and flutter (15). In addition to thyroid function abnormalities, patients diagnosed with GD may have abnormal thyroid antigens and antibodies (16, 17). Moreover, individuals with GD have a significantly higher risk for other autoimmune diseases, including T1D (18), rheumatoid arthritis (19), and systemic lupus erythematosus (20), than the general population. Previous studies have reported a correlation between GD and T1D (18, 21, 22); however, few studies have examined bidirectional causality. Thus, it is crucial to demonstrate a causal link between GD and T2D.

Mendelian randomization (MR) analysis is a novel research method that has emerged over the last decade. MR analysis is superior to randomized controlled trials in several respects. First, MR analysis uses genetic variation (single-nucleotide polymorphisms [SNPs]) as a random assignment to remove the effects of confounding factors and reverse causality, enabling the examination of the cause–effect relationship between exposure and outcome (23–26). Additionally, compared with traditional randomized group experiments, MR can save time and reduce economic costs while obtaining a larger sample size.

To identify any potential bidirectional causal relationship between GD, T1D, and T2D, this study used genome-wide association study (GWAS) data from publicly accessible databases in a bidirectional two-sample analysis. Furthermore, we clarified the causal relationship between TSH, TPO, and Tg and T1D and T2D through multifactorial MR analysis. This study offers a novel approach to the prevention of GD and DM.

## 2 Materials and methods

### 2.1 Study design

**Figure 1:**
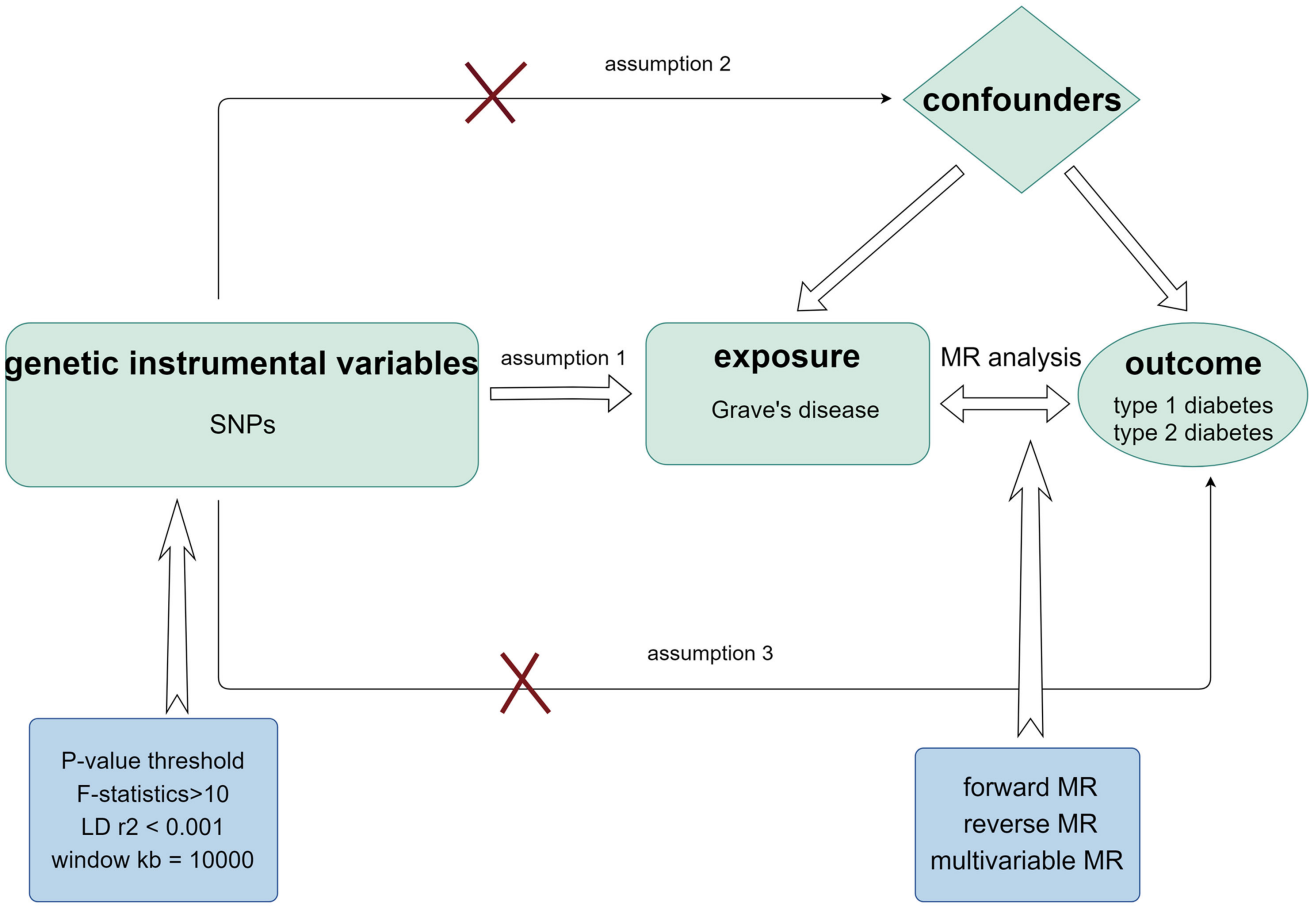
Schematic diagram of MR analysis satisfying three key assumptions. SNP, Single nucleotide polymorphism.

A bidirectional two-sample MR study was performed to determine the cause–effect relationship between GD and T1D. We also performed multivariate MR (MVMR) analysis to reduce the interference of confounding variables, such as TPO, TSH, and Tg, on the results. The research design process is illustrated in **Figure 1**.

### 2.2 GWAS data sources and selection of instrumental variables

Utilizing genetic IVs associated with GD obtained from a study by Sakaue et al. (27), this study investigated 220 human phenotypes, including thyroid dysfunction, in a European population with a large sample size of 458,620. Genetic data associated with TPO, Tg, and TSH were acquired from a study carried out by Benjamin et al. with a sample size of 3301 (28). Genetic data associated with GD, TPO, Tg, and TSH can be found in the IEU Open GWAS database (https://gwas.mrcieu.ac.uk/). To render the selected IVs for MR analysis comprehensive, three assumptions must be made: (1) The SNPs utilized as IVs should have a high correlation with exposure. Therefore, maintaining a clear logical structure when presenting these points is essential. (2) Genetic variation has no bearing on the possible genetic or environmental factors that could influence the outcome. (3) IVs should only affect the outcomes through exposure (29, 30). Subsequently, we determined the following selection criteria to reduce the likelihood of missing SNPs while maintaining a high correlation with exposure: P-value< 5×10^-8^, linkage disequilibrium (LD) r^2^< 0.001, and window kb = 10,000. The SNPs used as IVs are listed in **Supplementary Tables 2–4**. F-statistics for every SNP were also produced to evaluate the usefulness of the IVs according to this formula:


$$F\;=\;\left(N-2\right)\times R^{2}/\left(1-R^{2}\right)$$

More precisely, a powerful instrument is typically indicated by an F-statistic greater than 10, which implies that IVs are predictive of the exposure variable (31). **Supplementary Table 1** provides detailed phenotypic information.

### 2.3 Genetic variants with a risk of T1D and T2D

To perform reverse MR analysis, it was necessary to extract SNPs related to T1D and T2D. GWAS data for T1D originated from a study conducted by Chiou et al. that encompassed a cohort of 520,580 individuals from the European population, including 18,942 cases and 501,638 controls (32). The associated data were obtained from https://gwas.mrcieu.ac.uk/. The GWAS information for T2D was gathered from the FinnGen biobank database (mailto:@online%7bfinngen), containing 29,193 cases and 182,573 controls (33). The assumptions and selection criteria mentioned previously are also applicable to the reverse MR analysis.

### 2.4 Two-sample MR analysis and sensitivity analysis

Three methods were used: inverse variance weighting (IVW), weighted median, and MR-Egger regression. Giving more weight to variants with smaller standard errors makes IVW a highly efficient method that provides precise and powerful estimates of causal effects (34). Therefore, IVW was regarded as the primary method. Scatter plots were constructed to visualize the MR results. The results were subjected to statistical tests and sensitivity studies to evaluate reliability. In a two-sample MR analysis, heterogeneity generally refers to discrepancies in effect estimates across different loci or studies. Heterogeneity among the SNPs in IVW computation was assessed using Cochran’s Q test (35). If the *P*-value was greater than 0.05, the assumption of existing heterogeneity among SNPs was rejected; otherwise, a random-effects model was used. Subsequently, the MR-Egger intercept test was performed to identify horizontal pleiotropic effects (36). If the *P*-value obtained by this test was< 0.05, the selected SNPs influenced multiple phenotypes. This means that a single genetic variant may affect more than one phenotype, and that these effects are not mediated by the primary exposure of interest. The MR-PRESSO test was used for global and distortion testing. The presence or absence of heterogeneity was determined using a global test. Heterogeneity was considered present if the results were statistically significant. Conversely, if the *P*-values for the global testing were greater than 0.05, heterogeneity was absent. The distortion test is used to identify outliers and determine whether the MR analysis results are affected (37); therefore, this test was used in the present study. Random-effect models were utilized to estimate the MR effect sizes and assess a meaningful cause–effect link between exposure and outcome. Finally, the total effect of each remaining SNP was estimated using the leave-one-out methodology (38). Funnel and forest plots were constructed to display the results more clearly and concisely.

### 2.5 Reverse MR analysis and sensitivity analysis

To determine whether there was a reverse causal relationship between higher genetic liability of T1D and T2D, and GD, we performed reverse MR analysis. The same genotypes and SNPs were used for consistency. We examined GD, T1D, and T2D as outcomes. Similar to the forward MR analysis, the SNPs for T1D and T2D were extracted using the criteria mentioned previously and are shown in **Supplementary Tables 3** and **4**.

### 2.6 MVMR analysis

As most patients with GD have comorbidities, such as abnormal serum TPO, Tg, and TSH levels, we conducted an MVMR analysis to identify whether these factors contribute to the heightened risk of developing T1D and T2D.

## 3 Results

### 3.1 IV selection

We selected the following SNPs associated with GD, T1D, and T2D according to previous criteria. There were 27, 88, and 55 SNPs associated with GD, T1D, and T2D, respectively. In addition, 15 outliers were found for GD, 8 for T1D, and 1 for T2D through the MR-PRESSO test. Supplementary all selected SNPs and MR-PRESSO results are displayed in **Supplementary Material**. The F-statistic for each SNP was > 10, suggesting a significant association between the SNPs and exposure.

### 3.2 Two-sample MR analysis

**Figure 2:**
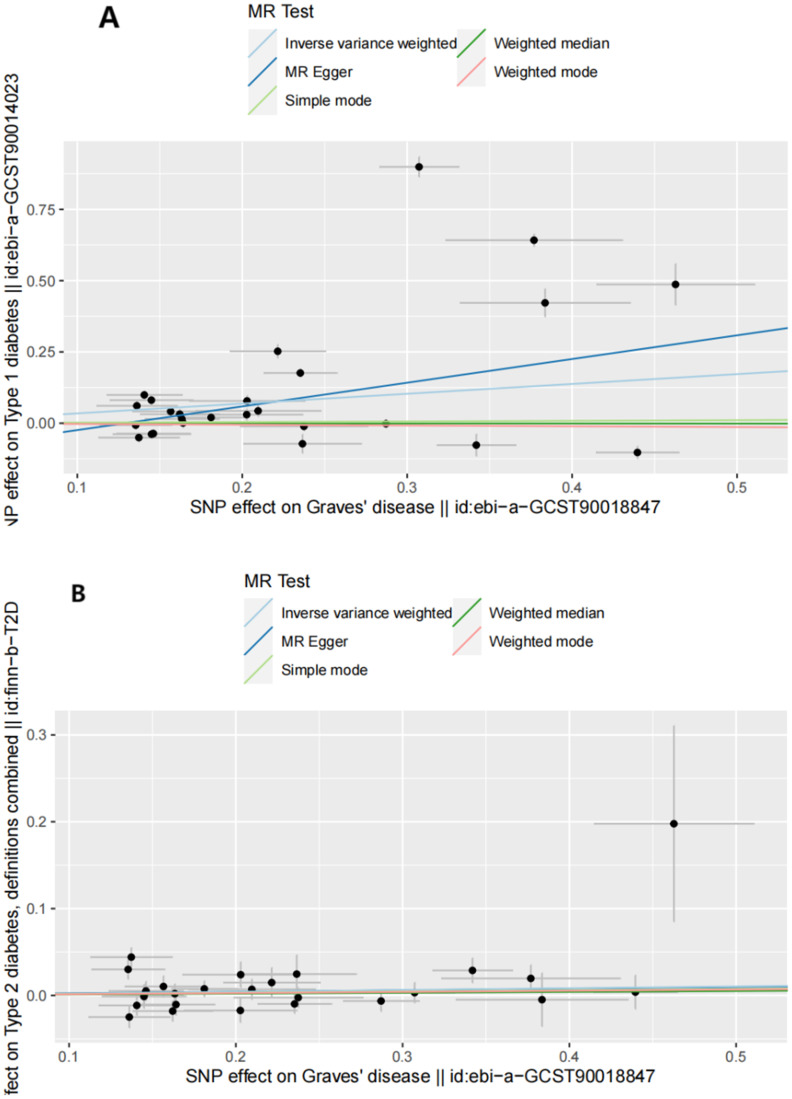
Scatter plots of forward MR analysis. **(A)** Scatter plots of SNP effects on GD and T1D risk. X axes represent SNP effects on GD. Y axes represent SNP effects on T1D risk. **(B)** Scatter plots of SNP effects on GD and T2D risk. X axes represent SNP effects on GD. Y axes represent SNP effects on T2D risk.

The IVW analysis results demonstrated that higher genetic liability of GD will increase risk of T1D (odds ratio [OR] (95% confidence interval [CI]) = 1.411 (1.077–1.848), *P* = 0.012). This suggests that the high genetic liability of GD is associated with a 41.1% increased risk of T1D. However, the weighted-median analysis results suggested no increased risk of T1D as genetic susceptibility to GD increases (OR[95% CI]= 0.998[0.927,1.074],*P*=0.949).The causal relationship between GD and T2D was also significant (OR (95% CI) = 1.059 (1.025–1.095), *P* = 5.53e-04). The elevated genetic liability of GD is potentially associated with a 5.9% increased risk of T2D. **Table 1** and **Figure 2** present the results of the MR analysis.

**Table 1:** Result of forward and reverse mendelian randomization analysis.

Bidirectional MR analysis			
Exposure-outcome	method	P-value	OR [95%CI]
**GD—T1D**	MR Egger	0.046	2.295[1.060,4.970]
	Weighted median	0.949	0.998[0.927,1.074]
	IVW	0.012	1.411[1.077,1.848]
**GD—T2D**	MR Egger	0.118	1.077[0.984, 1.179
	Weighted median	0.012	1.028[1.006,1.050]
	IVW	5.53e-04	1.059[1.025,1.095]
**T2D—GD**	MR Egger	0.798	0.968[0.757,1.238]
	Weighted median	0.933	0.994[0.874,1.131]
	IVW	0.468	0.963[0.869,1.066]
**T1D—GD**	MR Egger	2.479e-04	1.124[1.059,1.194]
	Weighted median	9.794e-37	1.192[1.160,1.224]
	IVW	1.913e-10	1.173[1.117,1.231]

GD, Grave’s Disease; T1D, type 1 diabetes; T2D, type 2 diabetes; IVW, inverse variance weighting.

### 3.3 Sensitivity analyses

**Figure 3:**
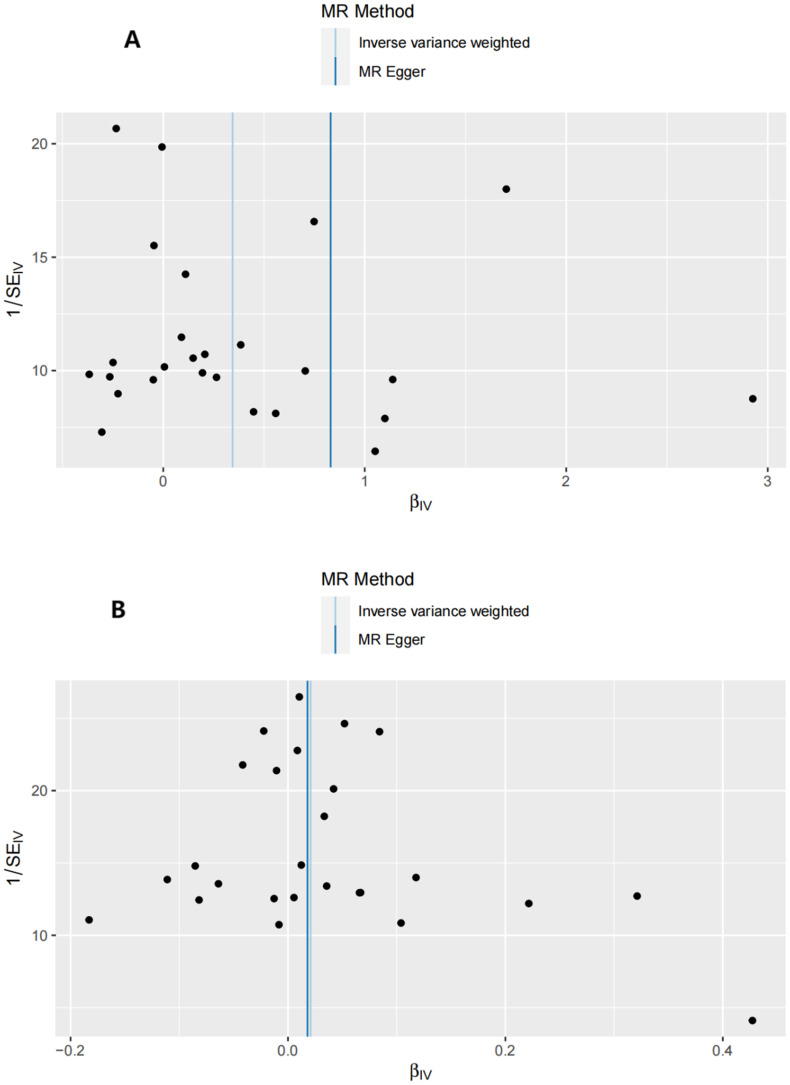
Funnel plots of the forward MR analysis. **(A)** exposure: GD, outcome: T1D. **(B)** exposure: GD, outcome: T2D.

**Figure 4:**
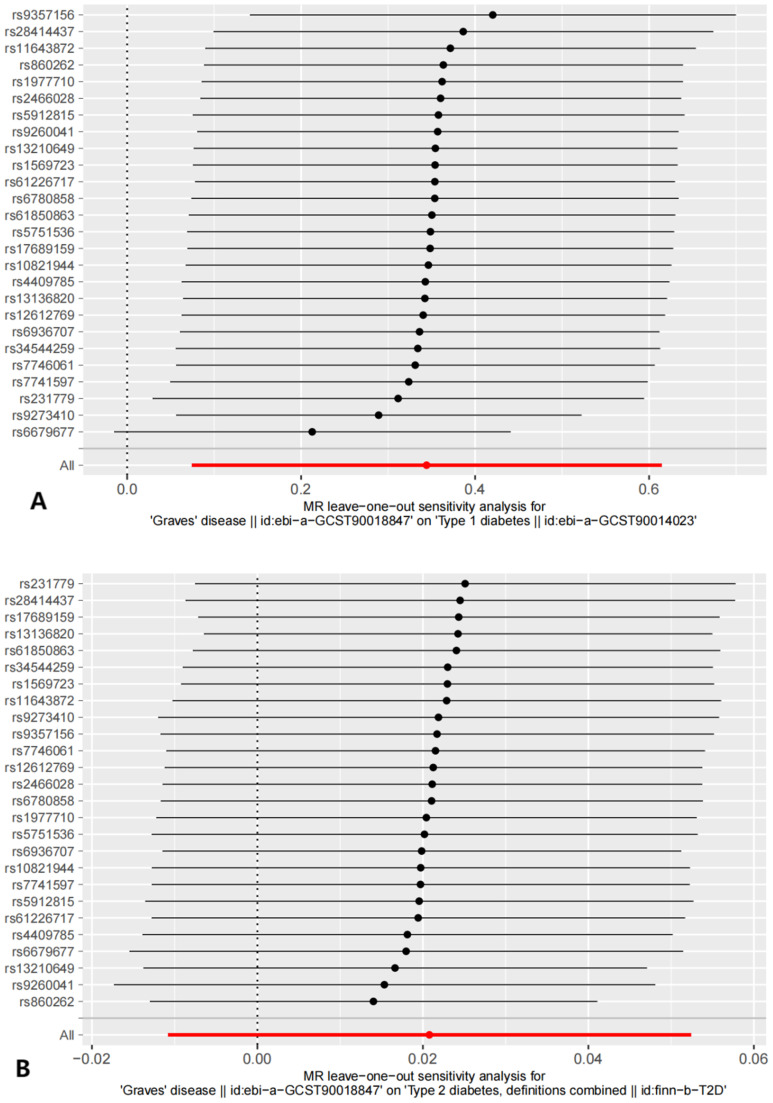
The results of Leave-one-out analysis. **(A)** leave-one-out analysis on causal relationship between GD and T1D. **(B)** leave-one-out analysis on causal relationship between GD and T2D.

Sensitivity analyses included the Cochran’s Q test and horizontal pleiotropy testing (**Table 2**). Heterogeneity was present in all analyses, as indicated by a P-value< 0.05. The MR-PRESSO test results showed that heterogeneity and outliers were present in all datasets. The MR effect magnitude was re-estimated using random-effects models, and causality was verified. The MR-Egger intercept tests of GD had *P* > 0.05, which suggests that horizontal pleiotropy was not significant (**Table 2**). The funnel plots for these analyses are shown in **Figure 3**. Ultimately, the leave-one-out analysis and visualization supported the reliability of our findings (**Figure 4**).

**Table 2:** Results of heterogeneity and horizontal pleiotropy in the bidirectional MR analysis.

Heterogeneity and pleiotropy test						
Exposure-outcome	Cochran’s Q Test				MR Egger intercept	
	Egger-Q Value	Pval-Egger	IVW-Q value	Pval-IVW	Intercept value	pval
**GD-T1D**	1621.986	0	1738.855	0	-0.107	0.201
**GD-T2D**	163.088	1.181e-22	164.114	2.013e-22	-0.004	0.701
**T1D-GD**	422.197	6.886e-47	432.656	2.269e-48	0.032	0.012
**T2D-GD**	98.391	1.524e-4	98.396	2.125e-4	-6.310e-4	0.962

GD, Grave’s Disease; T1D, type 1 diabetes; T2D, type 2 diabetes; IVW, inverse variance weighting.

### 3.4 Reverse MR analysis and sensitivity analysis

**Figure 5:**
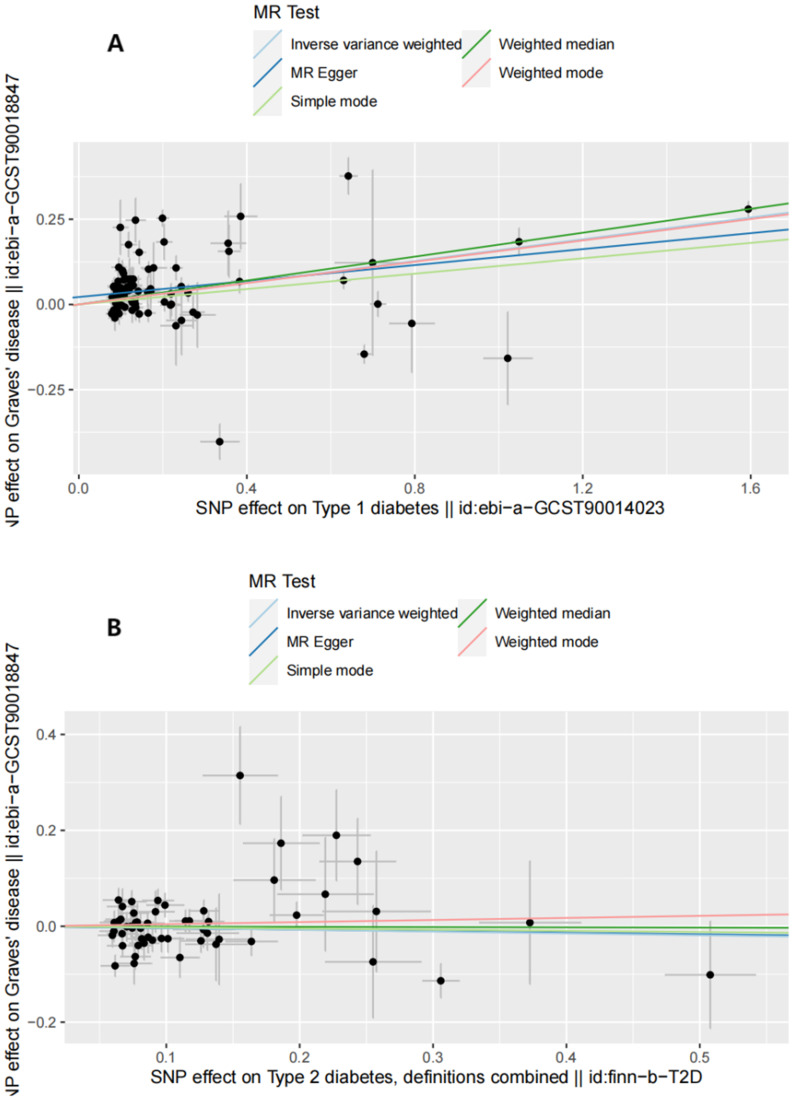
Scatter plots of reverse MR analysis. **(A)** Scatter plots of SNP effects on T1D and GD risk. X axes represent SNP effects on T1D. Y axes represent SNP effects on GD risk. **(B)** Scatter plots of SNP effects on T2D and GD risk. X axes represent SNP effects on T2D. Y axes represent SNP effects on GD risk.

**Figure 6:**
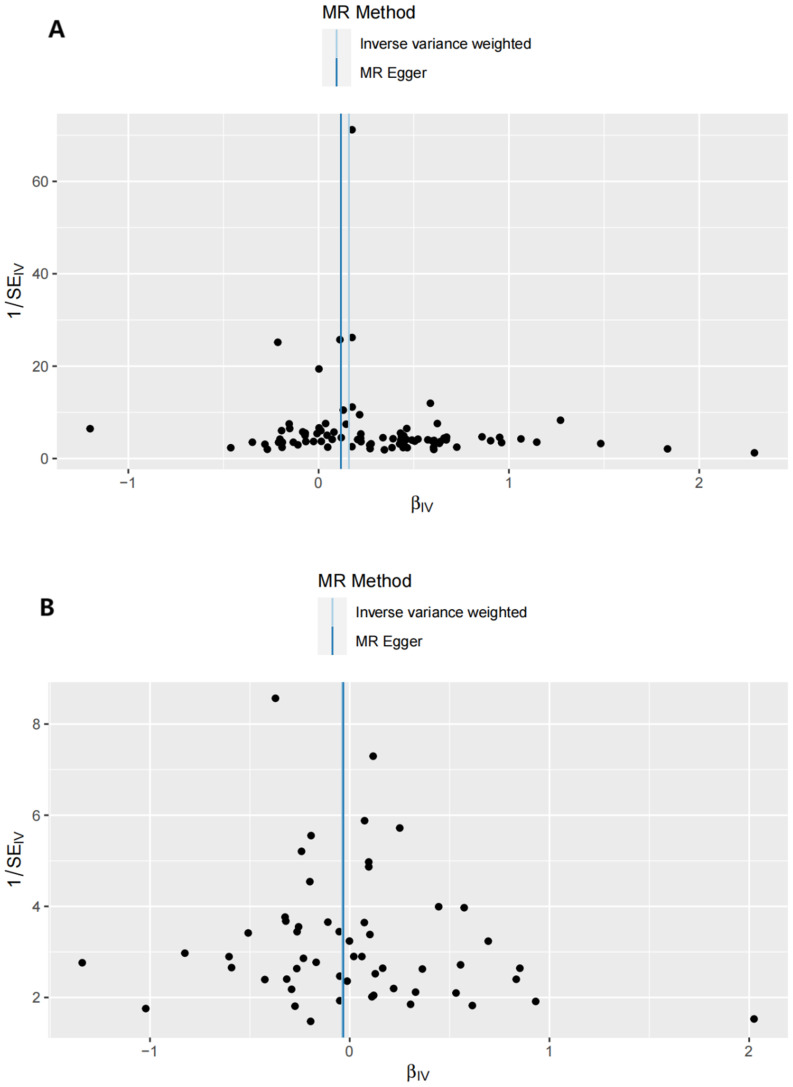
Funnel plots of the reverse MR analysis. **(A)** exposure: T1D, outcome: GD. **(B)** exposure: T2D, outcome: GD.

**Figure 7:**
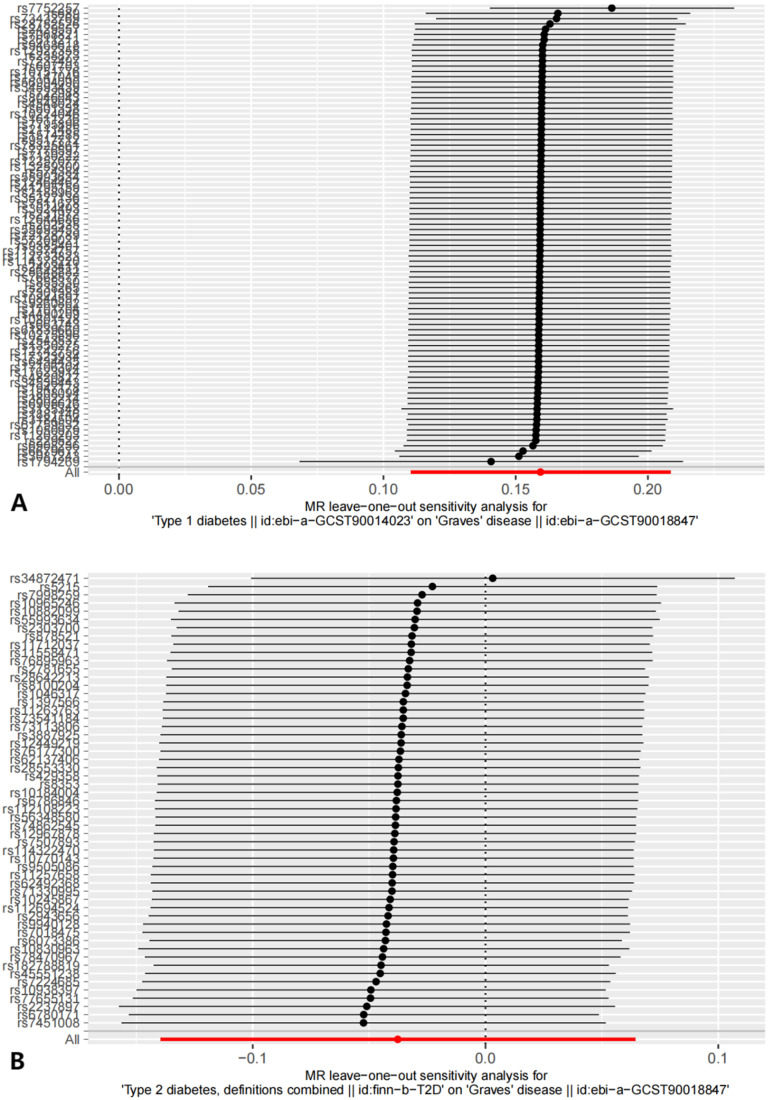
The results of Leave-one-out analysis on reverse MR analysis. **(A)** leave-one-out analysis on causal relationship between T1D and GD. **(B)** leave-one-out analysis on causal relationship between T2D and GD.

The reverse MR analysis and sensitivity analysis results shown in **Table 1** and **Figure 5** revealed the presence of a causal connection between the increased genetic liability of T1D and risk of GD [IVW, OR (95% CI) = 1.173(1.117,1.231), P = 1.913e-10]. In contrast, genetic susceptibility to T2D did not increase the risk of developing GD (OR (95% CI) = 0.963 (0.870–1.066), *P* = 0.468). **Table 2** displays the sensitivity analysis results of the reverse MR analysis. The funnel and leave-one-out plots of the reverse MR are shown in **Figures 6** and **7**, respectively.

### 3.5 MVMR analysis

**Table 3** demonstrates the causal links between the higher genetic liability of GD and T1D and the increased risk of T2D. In comparison, no causal relationship was observed between Tg, TSH, or TPO and T1D or T2D.

**Table 3:** results of MVMR analysis between DM and indicators for thyroid function.

Multivariable MR analysis		
Exposure-outcome	pval	OR [95%CI]
GD-T1D	0.0093	1.471[1.099,1.969]
Tg -T1D	0.392	2.584[0.293,22.770]
GD-T1D	0.013	1.429[1.078,1.895]
TSH-T1D	0.747	1.257[0.314,5.022]
GD-T1D	0.010	1.441[1.093,1.901]
TPO-T1D	0.498	0.732[0.297 1.806]
GD-T2D	0.364	1.015[0.982,1.050]
Tg -T2D	0.241	0.854 0.656 1.112
GD-T2D	0.240	1.020[0.987,1.054]
TPO-T2D	0.783	0.987[0.899,1.083]
GD-T2D	0.222	1.021[0.987,1.056]
TSH-T2D	0.313	0.917[0.775,1.085]

GD, Grave’s Disease; T1D, type 1 diabetes; T2D, type 2 diabetes; Tg, thyroglobulin; TSH, thyroid stimulating hormone; TPO, thyroid peroxidase.

## 4 Discussion

GD and T2D were causally linked in this study, indicating that a genetic predisposition to GD increases the risk of T2D. Conversely, there was no significant causal relationship between GD and T1D. However, reverse MR suggested that genetic liability to T1D increases the risk of developing GD, while T2D does not. Furthermore, there is inadequate evidence to suggest that abnormal TSH, TPO, and Tg levels increase the risk of developing T1D or T2D in patients with GD.

We believe that the increased risk of developing DM due to GD can be explained by certain aspects, such as the commonality of autoimmune mechanisms. GD is a chronic autoimmune thyroid disorder characterized by an abnormal immune response to thyroid antigens. Several animal studies have described a correlation between GD and regulatory T cells (Tregs) (39, 40). Some studies have reported varying degrees of functional impairment, decreased Treg cell efficiency, and enrichment of T helper 17 cells (Th17) in patients with GD (41–43). Th17 cells play a role in promoting inflammation and immune responses, whereas Tregs act as immunosuppressors. There may be a link between the onset and progression of DM (types 1 and 2) and an imbalance between Tregs and Th17 cells (43–45). A population-based study conducted in Finland revealed a significant correlation between T1D and other autoimmune diseases, including GD (46). The study found a significant association between hyperthyroidism and an increased risk of developing T1D (OR 2.98 [2.27–3.90]). In addition, GD has been identified as a primary cause of hyperthyroidism (47). In a separate study, it was found that out of 491 patients diagnosed with T1D, 122 tested positive for the TPO antibody, including 15 with autoimmune thyroid disease (48). In addition, a study of 500 individuals reported that patients with autoimmune thyroid disorders were more likely to develop other autoimmune diseases (49). Among them, researchers found T1D in 3.1% of patients with GD, confirming our finding that genetic susceptibility to GD increases the risk of developing T1D. Although several previous studies have suggested a potential correlation between GD and T1D, our findings indicate that increased genetic susceptibility to GD may not be a primary cause of the elevated risk of developing T1D. Considering the results of the IVW analysis are inconsistent with those of the weighted-media analysis, we conclude that the causal relationship between GD and T1D derived from the IVW analysis is unreliable. The disparate conclusions reached in this study in comparison to those of previous studies may be attributable to the presence of unavoidable confounding variables. To this end, larger sample sizes from other databases should be included in subsequent studies to validate the aforementioned conclusions.

The onset and progression of T2D is a complex process involving multiple mechanisms. Several studies have reported on the involvement of immune factors in the pathogenesis of this disease. Obesity-associated chronic inflammation is an important factor in the predisposition to T2D (44, 50). Multiple studies have shown that an imbalance between Treg and Th17 cells, specifically a deficiency of Tregs and an excess of Th17 cells, is linked to obesity, insulin resistance, and T2D (51–53). Some studies have suggested that systemic Treg defects are associated with diabetic nephropathy, retinopathy, and diabetic foot ulcers (54, 55). An increasing number of Tregs in the body has been shown to improve the severity of diabetes in some animal studies. Fengjie et al. discovered that Tregs can promote the mobilization of endothelial progenitor cells, anti-inflammatory effects, and cytoprotection (56). This can inhibit vascular endothelial hyperplasia and alleviate the vascular degeneration caused by elevated blood glucose levels in diabetic pigs. One study found that CD4+ Tregs decreased in the visceral tissues of patients with T2D (57). This suggests that an imbalance between the quality and capability of Treg and Th17 cells is involved in the development of GD, T1D, and T2D. This finding is consistent with the bidirectional causal relationship between GD and T1D. In contrast, the etiology of T2D is multifactorial, with immunological factors being one of the many contributors to its onset or progression. Consequently, patients with GD may have an increased risk of T2D due to the disruption of immune system homeostasis, while T2D does not necessarily increase the risk of GD.

Chronic inflammation should also be taken into consideration. It is common for autoimmune diseases to be accompanied by a chronic inflammatory response (58–61). Researchers have revealed that some cytokines, including transforming growth factor beta (TGF-β), interleukin-6 (IL-6), IL-4, IL-5, IL-10, IL-13, IL-1β, and interferon-gamma (IFN-γ), are associated with GD. T1D is associated with chronic inflammation (62), and several studies have reported that cytokines, such as IFN-γ, tumor necrosis factor (TNF)-α, and IL-1β, contribute to the pathogenesis of T1D (63–65). We speculate that chronic inflammation triggered by GD may contribute to the development of T1D, and vice versa. This finding suggests a bidirectional causal relationship between GD and T1D. In recent research, T2D has been increasingly associated with chronic inflammation (66). Chronic inflammation with increased levels of various cytokines contributes to the development of T2D. It has been reported that conventional type 1 dendritic cells can promote insulin resistance by increasing IFN-γ production (67). IL1-β, IL-6, IL-8, TNF-α, NF-κB, and MAPK were also significantly increased in the T2D and prediabetes groups compared to non-diabetic groups, indicating their role as pro-inflammatory factors (68). Chronic inflammation resulting from GD-induced increases in the levels of inflammatory factors, such as IFN-γ, IL-1β, and TNF-α *in vivo*, may contribute to insulin resistance and increase the risk of developing T2D by affecting insulin sensitivity. It is essential to note that this suggestion is based on objective evidence rather than subjective evaluation. Chronic inflammation is not the sole factor in the development of T2D. Therefore, the complex etiology of T2D indicates that GD development is not necessarily increased by T2D.

Similar to other studies, this study has certain limitations. First, database limitations prevented the inclusion of indicators related to thyroid function, such as thyroid hormone, TSH receptor, anti-Tg, and anti-TPO. Additional research is required to examine the relationships between other indicators and DM. Second, our study did not investigate the causal relationship between Hashimoto’s thyroiditis and T1D or T2D. Future studies should explore autoimmune thyroiditis. Third, the scope of this study was restricted to the European population. Forth, due to the limitation of the database, the sample of MVMR is too small. The follow-up study will incorporate a larger sample size and disease-related exposure into the MVMR portion of the study. Therefore, additional research is necessary to verify whether our findings are applicable to other research groups.

## 5 Conclusion

In the present study, there was no increased risk of T1D as genetic susceptibility to GD increased, although higher genetic susceptibility to T1D has been shown to be associated with increased risk of developing GD. No significant causal relationship between TSH, TPO, or Tg and T1D or T2D was found in the MVMR analysis. According to the results mentioned before, higher genetic susceptibility to GD will increase the risk of T2D. The findings provide new insights into the control of DM.

## Data Availability

The original contributions presented in the study are included in the article/**Supplementary Material**. Further inquiries can be directed to the corresponding author/s.
