# Supplementary material for: Evaluating the causal effects between Grave’s disease and diabetes mellitus: a bidirectional Mendelian randomization study
Source: Front Endocrinol (Lausanne). 2024 Nov 6;15:1420499. doi: 10.3389/fendo.2024.1420499 (PMC11576183; doi:10.3389/fendo.2024.1420499)
Supplement: Supplementary file 6 [file DataSheet6.docx]

## Codes of Mendelian Randomization study

exp<-extract_instruments(outcomes = "finn-b-T2D")

outcome_dat<-extract_outcome_data(exp$SNP,outcomes = "ebi-a-GCST90018847")

dat<-harmonise_data(exposure_dat = exp,outcome_dat = outcome_dat)

mr(dat)

generate_odds_ratios(mr_res = mr(dat))

run_mr_presso(dat,NbDistribution = 3000)

mr_scatter_plot(mr_results = mr(dat),dat)

mr_heterogeneity(dat)

mr_funnel_plot(singlesnp_results = mr_singlesnp(dat))

mr_pleiotropy_test(dat)

mr_leaveoneout_plot(leaveoneout_results = mr_leaveoneout(dat))

## Codes of multivariate Mendelian Randomization study

id_exposure<-c("ebi-a-GCST90018847","prot-a-530")

id_outcome<-"finn-b-T2D"

exposure_dat<-mv_extract_exposures(id_exposure)

outcome_dat<-extract_outcome_data(exposure_dat$SNP,id_outcome)

mvdat<-mv_harmonise_data(exposure_dat,outcome_dat)

res<-mv_multiple(mvdat)

res_OR<-generate_odds_ratios(res$result)

res_OR
