# Supplementary material for: Evaluating the causal effects between Grave’s disease and diabetes mellitus: a bidirectional Mendelian randomization study
Source: Front Endocrinol (Lausanne). 2024 Nov 6;15:1420499. doi: 10.3389/fendo.2024.1420499 (PMC11576183; doi:10.3389/fendo.2024.1420499)
Supplement: Supplementary file 7 [file DataSheet7.docx]

**STROBE-MR checklist of recommended items to address in reports of Mendelian randomization studies**^1^ ^2^

| **Item No.** | **Section** | **Checklist item** | **Page No.** | **Relevant text from manuscript** |
| --- | --- | --- | --- | --- |
| 1 | **TITLE and ABSTRACT** | Evaluating the causal effects between Grave’s disease and diabetes mellitus: a bidirectional Mendelian randomization study  Running Title: relationships between Grave’s disease and diabetes mellitus |  |  |
|  | **INTRODUCTION** |  |  |  |
| 2 | **Background** | Graves’ disease (GD) is an autoimmune disease associated with an increased incidence of other autoimmune diseases. some studies have demonstrated that immunology also contributes to the pathophysiology of diabetes mellitus (DM). Given that immune factors are involved in the pathophysiology of both GD and DM, it seems plausible to suggest that there may be a causal relationship between them. MR analysis is superior to randomized controlled trials in several respects. First, Mendelian randomization (MR) analysis can remove the effects of confounding factors and reverse causality, enabling the examination of the cause-effect relationship between exposure and outcome. Secondly, MR can also save time and reduce economic costs while obtaining a larger sample size. To investigate the causality between GD and Diabetes mellitus (DM), we designed bidirectional two-sample MR and multivariable MR (MVMR) studies. |  |  |
| 3 | **Objectives** | The objective of this study was twofold. First, we sought to investigate the potential causal relationship between GD and type 1(T1D) and type 2 diabetes (T2D) through a bidirectional two-sample MR study. Second, we aimed to ascertain whether abnormal thyroid function indicators are responsible for the development and progression of (T1D) and (T2D) through a multivariable MR(MVMR) study. |  |  |
|  | **METHODS** |  |  |  |
| 4 | **Study design and data sources** | Present key elements of the study design early in the article. Consider including a table listing sources of data for all phases of the study. For each data source contributing to the analysis, describe the following: |  |  |
|  | a) | Setting: Study design: A bidirectional two-sample MR study was performed to determine the cause-effect relationship between GD and T1D. We also performed multivariate MR (MVMR) analysis to reduce the interference of confounding variables, such as TPO, TSH, and Tg, on the results. This study is based on a European population. The research design process is illustrated in Figure 1.  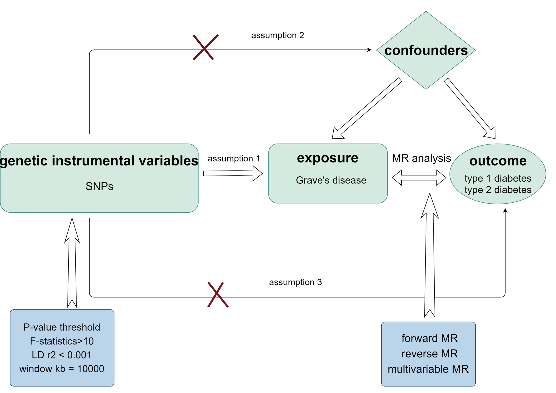 |  |  |
|  | b) | Participants:  Utilizing genetic IVs associated with GD investigated 220 human phenotypes with a large sample size of 458,620. Genetic data associated with TPO, Tg, and TSH were acquired from a study carried out by Benjamin et al. with a sample size of 3301. Genetic data associated with GD, TPO, Tg, and TSH can be found in the IEU Open GWAS database (<https://gwas.mrcieu.ac.uk/>).  GWAS data for T1D originated from a study conducted by Chiou et al. that encompassed a cohort of 520,580 individuals from the European population, including 18,942 cases and 501,638 controls. The associated data were obtained from <https://gwas.mrcieu.ac.uk/>. The GWAS information for T2D was gathered from the FinnGen biobank database ([mailto:@online{finngen),](mailto:@online%7bfinngen,) containing 29,193 cases and 182,573 controls  sources of data for all phases of the study：[supplementary\supplementary table1.csv](supplementary/supplementary%20table1.csv) |  |  |
|  | c) | we determined the following selection criteria to reduce the likelihood of missing SNPs while maintaining a high correlation with exposure: P-value < 5×10^-8^, linkage disequilibrium (LD) r^2^ < 0.001, and window kb = 10,000. The SNPs used as IVs are listed in Supplementary Tables 2–4. F-statistics for every SNP were also produced to evaluate the usefulness of the IVs according to this formula:  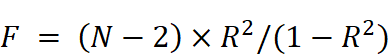  More precisely, a powerful instrument is typically indicated by an F-statistic greater than 10, which implies that IVs are predictive of the exposure variable |  |  |
|  | d) | Diagnostic criteria for diseases  GD, an autoimmune disorder primarily affecting the thyroid gland, is diagnosed through a meticulous assessment incorporating various clinical and laboratory criteria. The diagnostic criteria commonly adhere to the following guidelines:  1. Clinical Manifestations:  - Presence of hyperthyroid symptoms, such as weight loss, palpitations, heat intolerance, tremors, and hyperactivity.  - Physical signs including diffuse goiter, ophthalmopathy (such as proptosis or lid retraction), and/or dermopathy (e.g., pretibial myxedema).  2. Thyroid Function Tests:  - Elevated serum levels of free thyroxine (T4) and triiodothyronine (T3), accompanied by suppressed levels of thyroid-stimulating hormone (TSH), reflecting hyperthyroidism.  - The presence of thyroid receptor antibodies, notably thyrotropin receptor antibodies (TRAb), further supports the diagnosis, aiding in distinguishing Graves' disease from other causes of hyperthyroidism.  3. Radioiodine Uptake Studies:  - Increased radioactive iodine uptake by the thyroid gland, typically exceeding 30% at 24 hours, confirms the hyperfunctioning nature of the thyroid gland.  4. Imaging Studies:  - Ultrasonography may reveal a diffusely enlarged thyroid gland with increased vascularity, corroborating the clinical suspicion.  5. Histopathological Examination (if indicated):  - Fine-needle aspiration biopsy may be performed in cases of diagnostic uncertainty or suspicion of thyroid nodules to rule out concurrent thyroid malignancy.  T1D, characterized by autoimmune destruction of pancreatic beta cells leading to absolute insulin deficiency, necessitates a thorough diagnostic evaluation incorporating clinical, laboratory, and immunological parameters. The diagnostic criteria for type 1 diabetes typically encompass the following elements:  1. Clinical Presentation:  - Manifestation of classical symptoms of hyperglycemia, including polyuria, polydipsia, unexplained weight loss, and, in some cases, diabetic ketoacidosis (DKA) characterized by nausea, vomiting, abdominal pain, and altered mental status.  2. Plasma Glucose Levels:  - Fasting plasma glucose levels ≥ 126 mg/dL (7.0 mmol/L) on two separate occasions, as measured after an overnight fast of at least 8 hours.  - Random plasma glucose levels ≥ 200 mg/dL (11.1 mmol/L) with accompanying symptoms of hyperglycemia, such as polyuria and polydipsia.  3. Hemoglobin A1c (HbA1c) Levels:  - HbA1c levels ≥ 6.5% (48 mmol/mol) on a standardized assay. Elevated HbA1c reflects chronic hyperglycemia and aids in the diagnosis and monitoring of glycemic control over the preceding 2-3 months.  4. Autoantibody Testing:  - Detection of autoantibodies directed against pancreatic beta cell antigens, including islet cell cytoplasmic autoantibodies (ICA), glutamic acid decarboxylase autoantibodies (GADA), insulinoma-associated-2 autoantibodies (IA-2A), and zinc transporter 8 autoantibodies (ZnT8A), indicative of autoimmune destruction of pancreatic beta cells.  5. C-Peptide Levels:  - Reduced or undetectable serum levels of C-peptide, a byproduct of endogenous insulin production, reflecting diminished beta cell function and insulin secretion.  Type 2 diabetes mellitus, a metabolic disorder characterized by insulin resistance and relative insulin deficiency, necessitates a methodical approach to diagnosis, integrating clinical, laboratory, and imaging modalities. The diagnostic criteria for type 2 diabetes typically encompass the following elements:  1. Fasting Plasma Glucose (FPG) Levels:  - Fasting plasma glucose levels ≥ 126 mg/dL (7.0 mmol/L) on two separate occasions, as measured after an overnight fast of at least 8 hours.  2. Oral Glucose Tolerance Test (OGTT):  - Two-hour plasma glucose levels ≥ 200 mg/dL (11.1 mmol/L) during an oral glucose tolerance test (OGTT), administered using a glucose load containing 75 grams of anhydrous glucose dissolved in water, performed under standardized conditions.  3. Hemoglobin A1c (HbA1c) Levels:  - HbA1c levels ≥ 6.5% (48 mmol/mol) on a standardized assay. HbA1c reflects average blood glucose levels over the preceding 2-3 months and is less affected by short-term fluctuations.  4. Random Plasma Glucose Levels with Symptoms:  - In individuals presenting with classic symptoms of hyperglycemia (e.g., polyuria, polydipsia, unexplained weight loss) or hyperglycemic crisis, a random plasma glucose level ≥ 200 mg/dL (11.1 mmol/L) confirms the diagnosis of diabetes.  5. Additional Testing and Considerations:  - Screening for diabetes-related complications, including retinopathy, nephropathy, and neuropathy, is essential at the time of diagnosis and periodically thereafter.  - Assessment of cardiovascular risk factors, lipid profile, blood pressure, and kidney function is integral for comprehensive management |  |  |
|  | e) | All participating studies of GWAS have obtained approval from relevant institutional review boards, and written informed consent was received from all subjects. |  |  |
| 5 | **Assumptions** | To render the selected IVs for MR analysis comprehensive, three assumptions must be made: (1) The SNPs utilized as IVs should have a high correlation with exposure. Therefore, maintaining a clear logical structure when presenting these points is essential. (2) Genetic variation has no bearing on the possible genetic or environmental factors that could influence the outcome. (3) IVs should only affect the outcomes through exposure. |  |  |
| 6 | **Statistical methods: main analysis** | Describe statistical methods and statistics used |  |  |
|  | a) | There are no quantitative variables in the analyses. |  |  |
|  | b) | we determined the following selection criteria to reduce the likelihood of missing SNPs while maintaining a high correlation with exposure: P-value < 5×10^-8^, linkage disequilibrium (LD) r^2^ < 0.001, and window kb = 10,000. The SNPs used as IVs are listed in Supplementary Tables 2–4. F-statistics for every SNP were also produced to evaluate the usefulness of the IVs according to this formula:  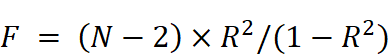  More precisely, a powerful instrument is typically indicated by an F-statistic greater than 10, which implies that IVs are predictive of the exposure variable |  |  |
|  | c) | Describe the MR estimator (e.g. two-stage least squares, Wald ratio) and related statistics. Detail the included covariates and, in the case of two-sample MR, whether the same covariate set was used for adjustment in the two samples |  |  |
|  | d) | The data utilized in the study were derived from the comprehensive GWAS study and did not exhibit any instances of missing values. Furthermore, during the screening process for instrumental variables, missing values are excluded from the study because they do not meet the selection criteria for instrumental variables. |  |  |
|  | e) | As most patients with GD have comorbidities, such as abnormal serum TPO, Tg, and TSH levels, we conducted an MVMR analysis to identify whether these factors contribute to the heightened risk of developing T1D and T2D. The TwoSampleMR package from the R software was used in this process to analyze the potential causal relationships. |  |  |
| 7 | **Assessment of assumptions** | We set the selection criteria: (1)P-value < 5×10-8 (2) linkage disequilibrium (LD) r2 < 0.001(3) and window kb = 10,000 (4)F-statistic greater than 10 |  |  |
| 8 | **Sensitivity analyses and additional analyses** | Heterogeneity among the SNPs in IVW computation was assessed using Cochran's Q test. If the P-value was greater than 0.05, the assumption of existing heterogeneity among SNPs was rejected; otherwise, a random-effects model was used. Subsequently, the MR-Egger intercept test was performed to identify horizontal pleiotropic effects. If the P-value obtained by this test was < 0.05, the selected SNPs influenced multiple phenotypes. The MR-PRESSO test was used for global and distortion testing. The presence or absence of heterogeneity was determined using a global test. Heterogeneity was considered present if the results were statistically significant. Conversely, if the P-values for the global testing were greater than 0.05, heterogeneity was absent. The distortion test is used to identify outliers and determine whether the MR analysis results are affected; therefore, this test was used in the present study. Finally, the total effect of each remaining SNP was estimated using the leave-one-out methodology  Reverse MR analysis and sensitivity analysis  To determine whether there was a reverse causal relationship between T1D, T2D, and GD, we performed reverse MR analysis. The same genotypes and SNPs were used for consistency. We examined GD, T1D, and T2D as outcomes. Similar to the forward MR analysis, the SNPs for T1D and T2D were extracted using the criteria mentioned previously and are shown in Supplementary Tables 3 and 4.  MVMR analysis  As most patients with GD have comorbidities, such as abnormal serum TPO, Tg, and TSH levels, we conducted an MVMR analysis to identify whether these factors contribute to the heightened risk of developing T1D and T2D. |  |  |
| 9 | **Software and pre-registration** |  |  |  |
|  | a) | Software: R4.3.2. package: TwoSampleMR |  |  |
|  | b) | The study protocol and details have been pre-registered in Nanchang University and Jilin University |  |  |
|  | **RESULTS** |  |  |  |
| 10 | **Descriptive data** |  |  |  |
|  | a) | The number of SNPs of GD, T1D, and T2D is 24189816, 59999551, and 16380433, respectively. A total of 27, 88, and 55 SNPs associated with GD, T1D, and T2D, respectively, were included in the study. |  |  |
|  | b) | Report summary statistics for phenotypic exposure(s), outcome(s), and other relevant variables (e.g. means, SDs, proportions) GWAS summary statistics: source and description/SNPs of GD/SNPs of T1D/SNPs of T2D: [supplementary\supplementary table1.csv](supplementary/supplementary%20table1.csv)[supplementary\supplementary table2.csv](supplementary/supplementary%20table2.csv)[supplementary\supplementary table3.csv](supplementary/supplementary%20table3.csv)[supplementary\supplementary table4.csv](supplementary/supplementary%20table4.csv) |  |  |
|  | c) | There are no meta-analyses of previous studies included in the data sources |  |  |
|  | d) | For two-sample MR:  i.  We have set the selection criteria:P-value < 5×10-8, linkage disequilibrium (LD) r^2^ < 0.001, and window kb = 10,000.  ii.  To reduce the number of individuals who overlap between the exposure and outcome studies, we chose data from different databases for exposure and outcome, respectively. In addition, these SNPs as exposure and outcome were from articles published by authors from different countries, respectively |  |  |
| 11 | **Main results** |  |  |  |
|  | a) | The p-value for each SNP is less than 0.05, and the F-statistic for each SNP is greater than 10. There were 27, 88, and 55 SNPs associated with GD, T1D, and T2D, respectively. |  |  |
|  | b) | \| Bidirectional MR analysis \| \| \| \| \| --- \| --- \| --- \| --- \| \| Exposure-outcome \| method \| P-value \| OR [95%CI] \| \| GD—T1D \| MR Egger \| 0.046 \| 2.295[1.060,4.970] \| \| Weighted median \| 0.949 \| 0.998[0.927,1.074] \| \| IVW \| 0.012 \| 1.411[1.077,1.848] \| \| GD—T2D \| MR Egger \| 0.118 \| 1.077[0.984, 1.179 \| \| Weighted median \| 0.012 \| 1.028[1.006,1.050] \| \| IVW \| 5.53e-04 \| 1.059[1.025,1.095] \| \| T2D—GD \| MR Egger \| 0.798 \| 0.968[0.757,1.238] \| \| Weighted median \| 0.933 \| 0.994[0.874,1.131] \| \| IVW \| 0.468 \| 0.963[0.869,1.066] \| \| T1D—GD \| MR Egger \| 2.479e-04 \| 1.124[1.059,1.194] \| \| Weighted median \| 9.794e-37 \| 1.192[1.160,1.224] \| \| IVW \| 1.913e-10 \| 1.173[1.117,1.231] \| |  |  |
|  | c) | 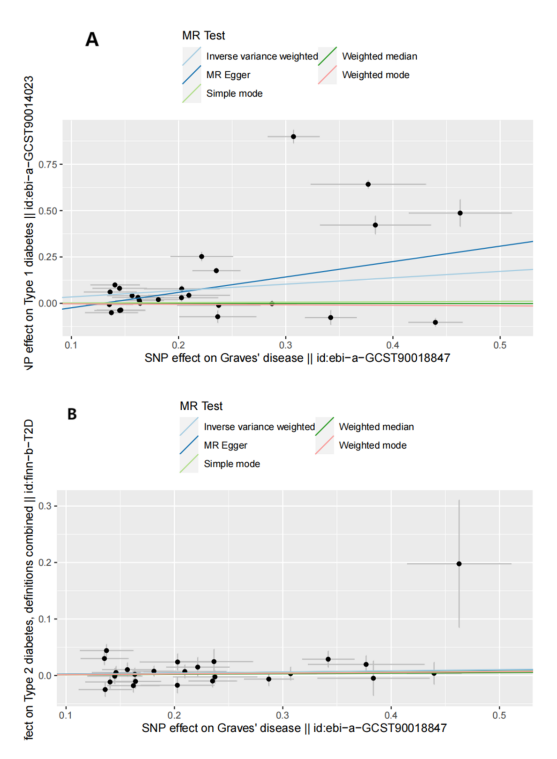  Results for forward MR analysis  A: exposure: GD Outcome: T1D  B: exposure: GD Outcome: T2D  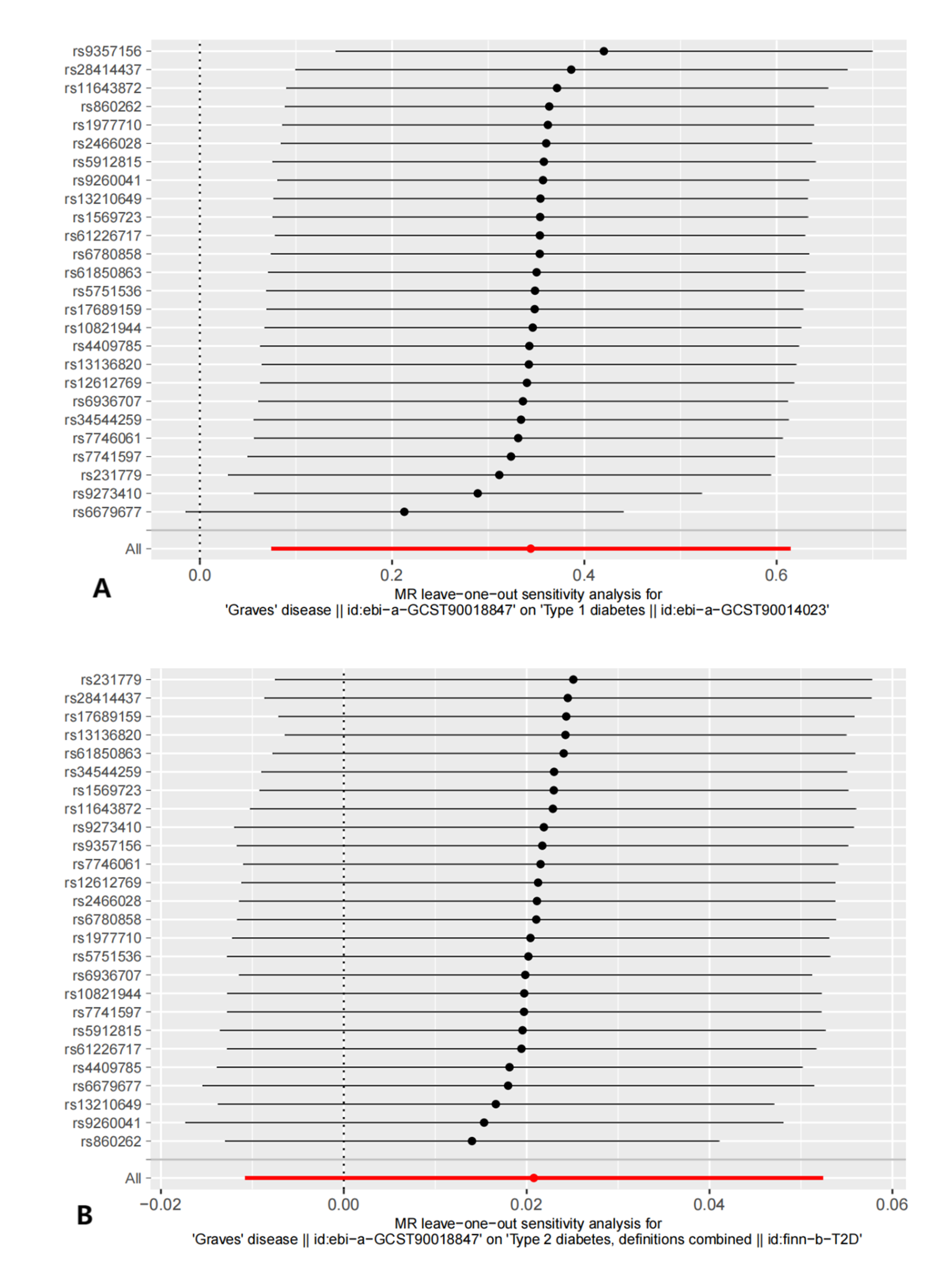  Forest plots for forward MR analysis  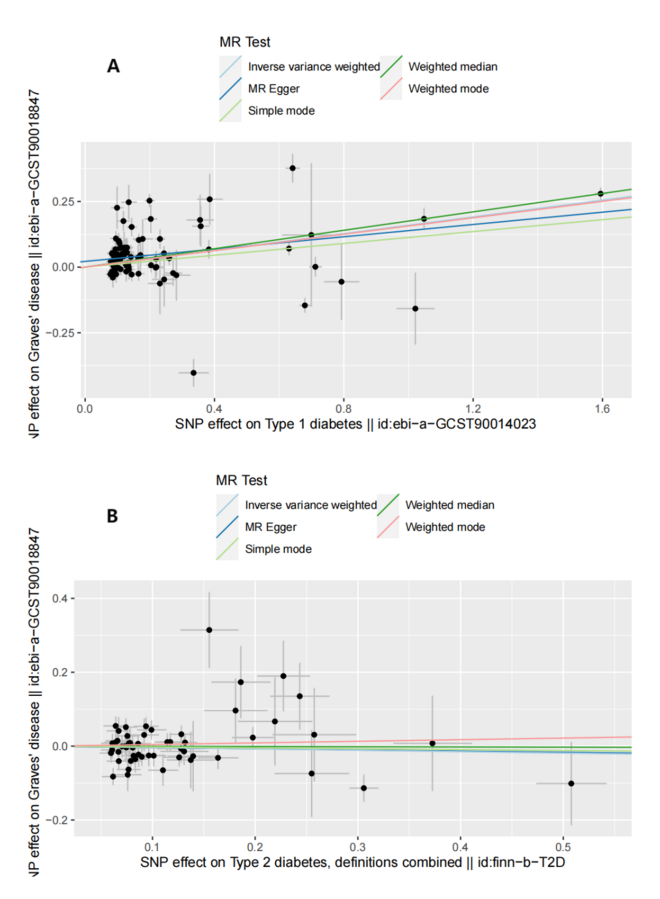  The results of reverse MR analysis  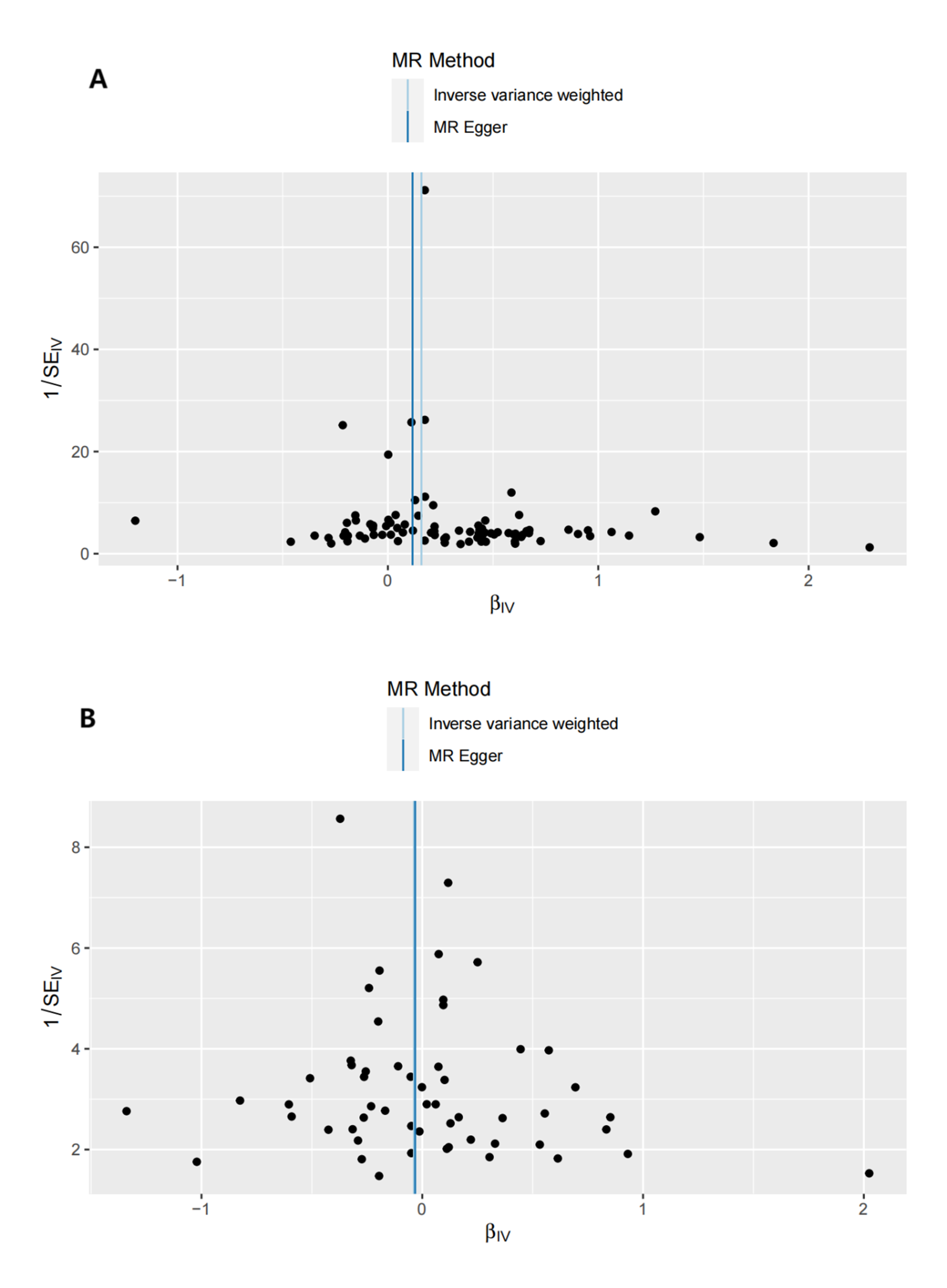  Funnel plots for reverse MR analysis  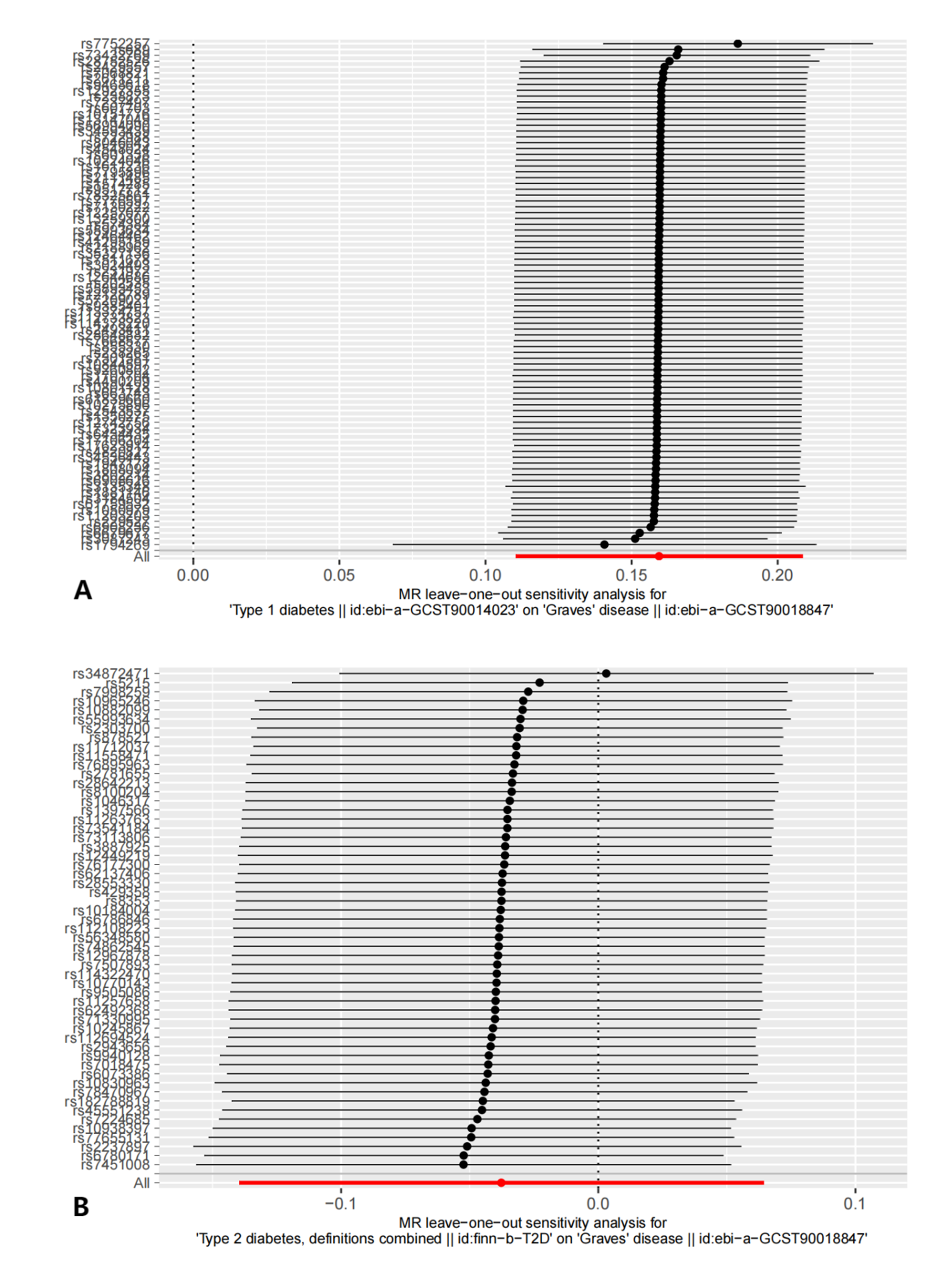  Forest plots for reverse MR analysis  Forest plots for reverse MR analysis  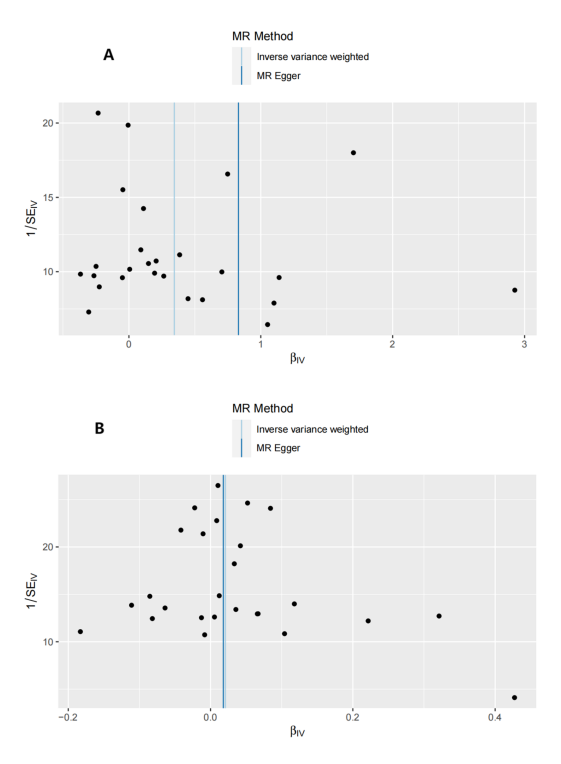  The funnel plots for forward MR analysis |  |  |
|  | d) | 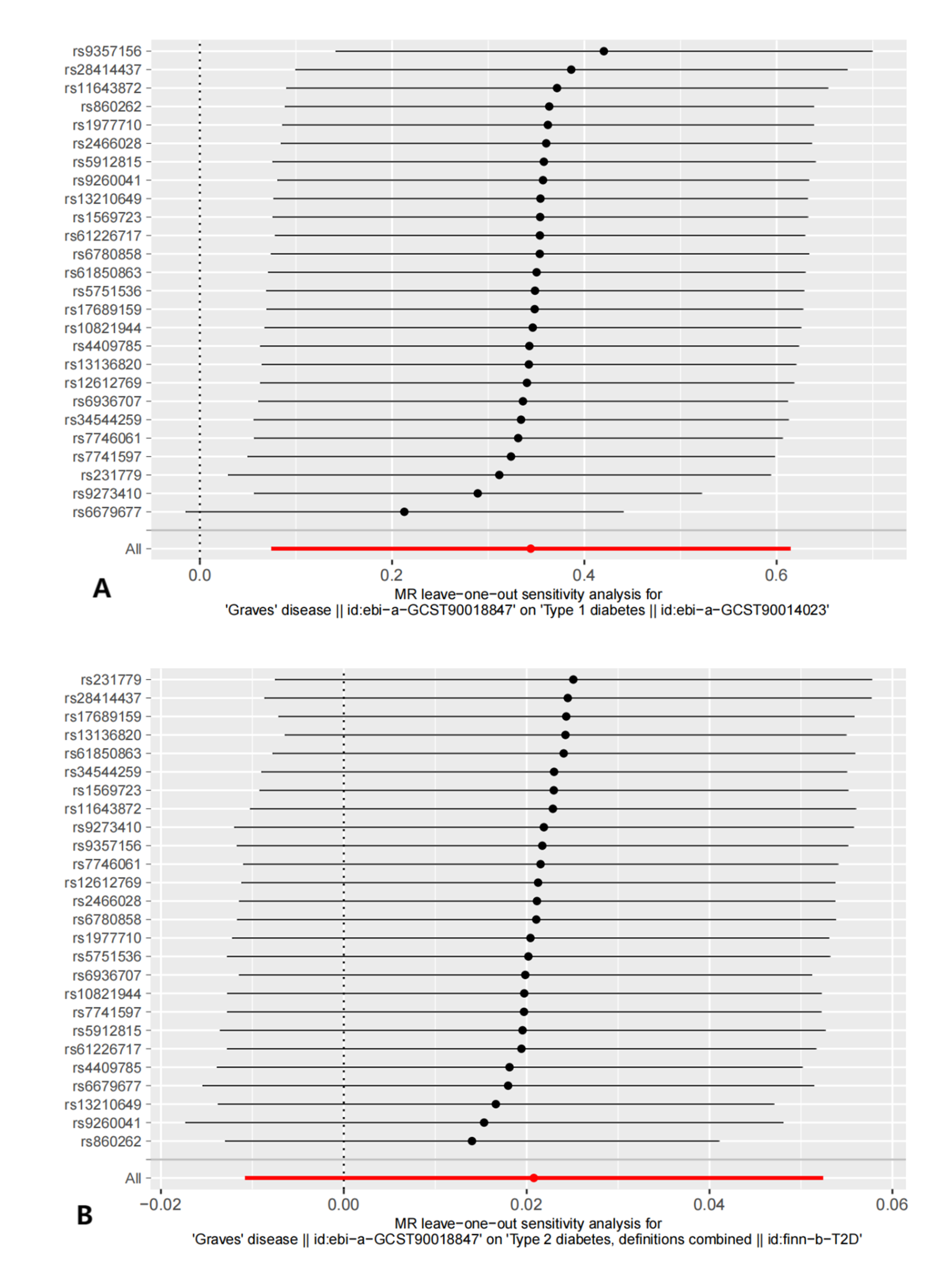  Forest plots for forward MR analysis  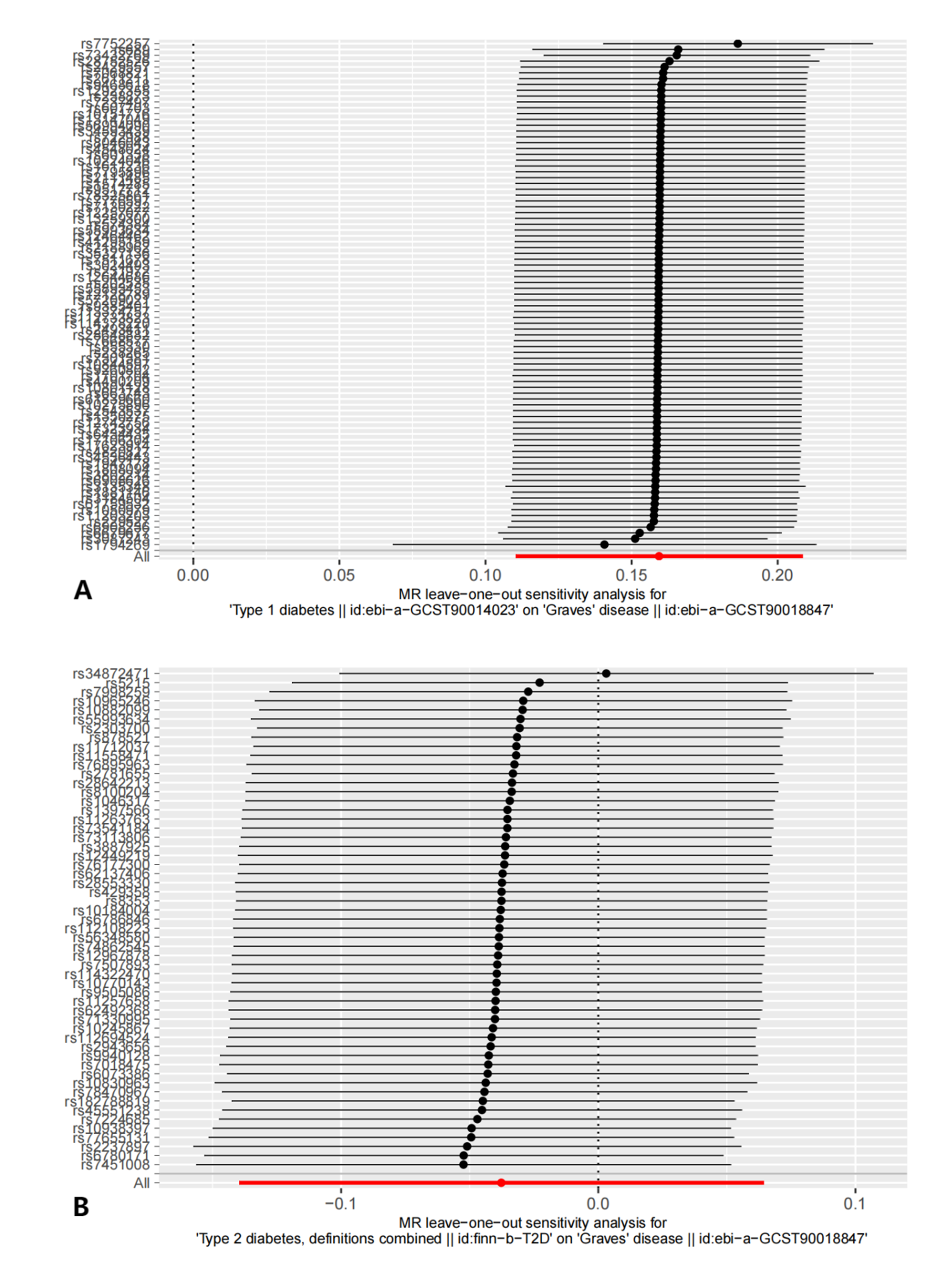  Forest plots for reverse MR analysis |  |  |
| 12 | **Assessment of assumptions** |  |  |  |
|  | a) | \| Bidirectional MR analysis \| \| \| \| \| --- \| --- \| --- \| --- \| \| Exposure-outcome \| method \| P-value \| OR [95%CI] \| \| GD—T1D \| MR Egger \| 0.046 \| 2.295[1.060,4.970] \| \| Weighted median \| 0.949 \| 0.998[0.927,1.074] \| \| IVW \| 0.012 \| 1.411[1.077,1.848] \| \| GD—T2D \| MR Egger \| 0.118 \| 1.077[0.984, 1.179 \| \| Weighted median \| 0.012 \| 1.028[1.006,1.050] \| \| IVW \| 5.53e-04 \| 1.059[1.025,1.095] \| \| T2D—GD \| MR Egger \| 0.798 \| 0.968[0.757,1.238] \| \| Weighted median \| 0.933 \| 0.994[0.874,1.131] \| \| IVW \| 0.468 \| 0.963[0.869,1.066] \| \| T1D—GD \| MR Egger \| 2.479e-04 \| 1.124[1.059,1.194] \| \| Weighted median \| 9.794e-37 \| 1.192[1.160,1.224] \| \| IVW \| 1.913e-10 \| 1.173[1.117,1.231] \| |  |  |
|  | b) | \| Heterogeneity and pleiotropy test \| \| \| \| \| \| \| \| --- \| --- \| --- \| --- \| --- \| --- \| --- \| \| Exposure-outcome \| Cochran’s Q Test \| \| \| \| MR Egger intercept \| \| \| Egger-Q Value \| Pval-Egger \| IVW-Q value \| Pval-IVW \| Intercept value \| pval \| \| GD-T1D \| 1621.986 \| 0 \| 1738.855 \| 0 \| -0.107 \| 0.201 \| \| GD-T2D \| 163.088 \| 1.181e-22 \| 164.114 \| 2.013e-22 \| -0.004 \| 0.701 \| \| T1D-GD \| 422.197 \| 6.886e-47 \| 432.656 \| 2.269e-48 \| 0.032 \| 0.012 \| \| T2D-GD \| 98.391 \| 1.524e-4 \| 98.396 \| 2.125e-4 \| -6.310e-4 \| 0.962 \| |  |  |
| 13 | **Sensitivity analyses and additional analyses** |  |  |  |
|  | a) | Sensitivity analyses included Cochran's Q test and horizontal pleiotropy testing (Table 2). Heterogeneity was present in all analyses, as indicated by a P-value < 0.05. The MR-PRESSO test results showed that heterogeneity and outliers were present in all datasets. The MR effect magnitude was re-estimated using random-effects models, and causality was verified. The MR-Egger intercept tests of GD had P > 0.05, which suggests that horizontal pleiotropy was not significant (Table 2). The funnel plots for these analyses are shown in Figure 3. Ultimately, the leave-one-out analysis and visualization supported the reliability of our findings (Figure 4). |  |  |
|  | b) | [supplementary\supplementary table 5.csv](supplementary/supplementary%20table%205.csv) |  |  |
|  | c) | GD and both T1D and T2D were causally linked in this study, indicating that a genetic predisposition to GD increases the risk of both T1D and T2D. However, reverse MR suggested that genetic susceptibility to T1D increases the risk of developing GD, while T2D does not. Furthermore, there is inadequate evidence to suggest that abnormal TSH, TPO, and Tg levels increase the risk of developing T1D or T2D in patients with GD. |  |  |
|  | d) | Some studies have reported varying degrees of functional impairment, decreased Treg cell efficiency, and enrichment of T helper 17 cells (Th17) in patients with GD. Th17 cells play a role in promoting inflammation and immune responses, whereas Tregs act as immunosuppressors. There may be a link between the onset and progression of DM (types 1 and 2) and an imbalance between Tregs and Th17 cells. A population-based study conducted in Finland revealed a significant correlation between T1D and other autoimmune diseases, including GD. The study found a significant association between hyperthyroidism and an increased risk of developing T1D (OR 2.98 [2.27–3.90]). In a separate study, it was found that out of 491 patients diagnosed with T1D, 122 tested positive for the TPO antibody, including 15 with autoimmune thyroid disease. In addition, a study of 500 individuals reported that patients with autoimmune thyroid disorders were more likely to develop other autoimmune diseases. Additionally， multiple studies have shown that an imbalance between Treg and Th17 cells, specifically a deficiency of Tregs and an excess of Th17 cells, is linked to obesity, insulin resistance, and T2D |  |  |
|  | e) | 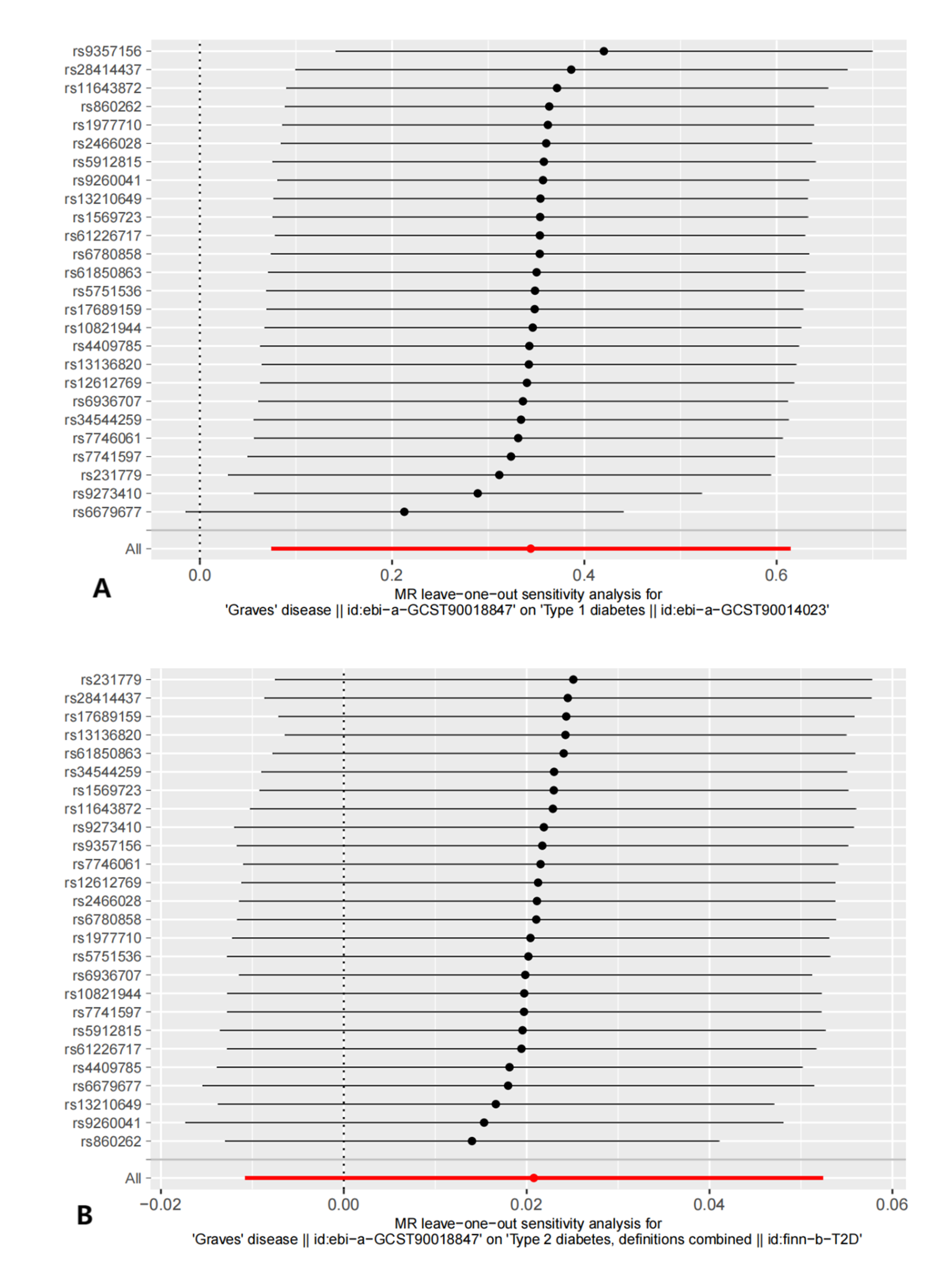 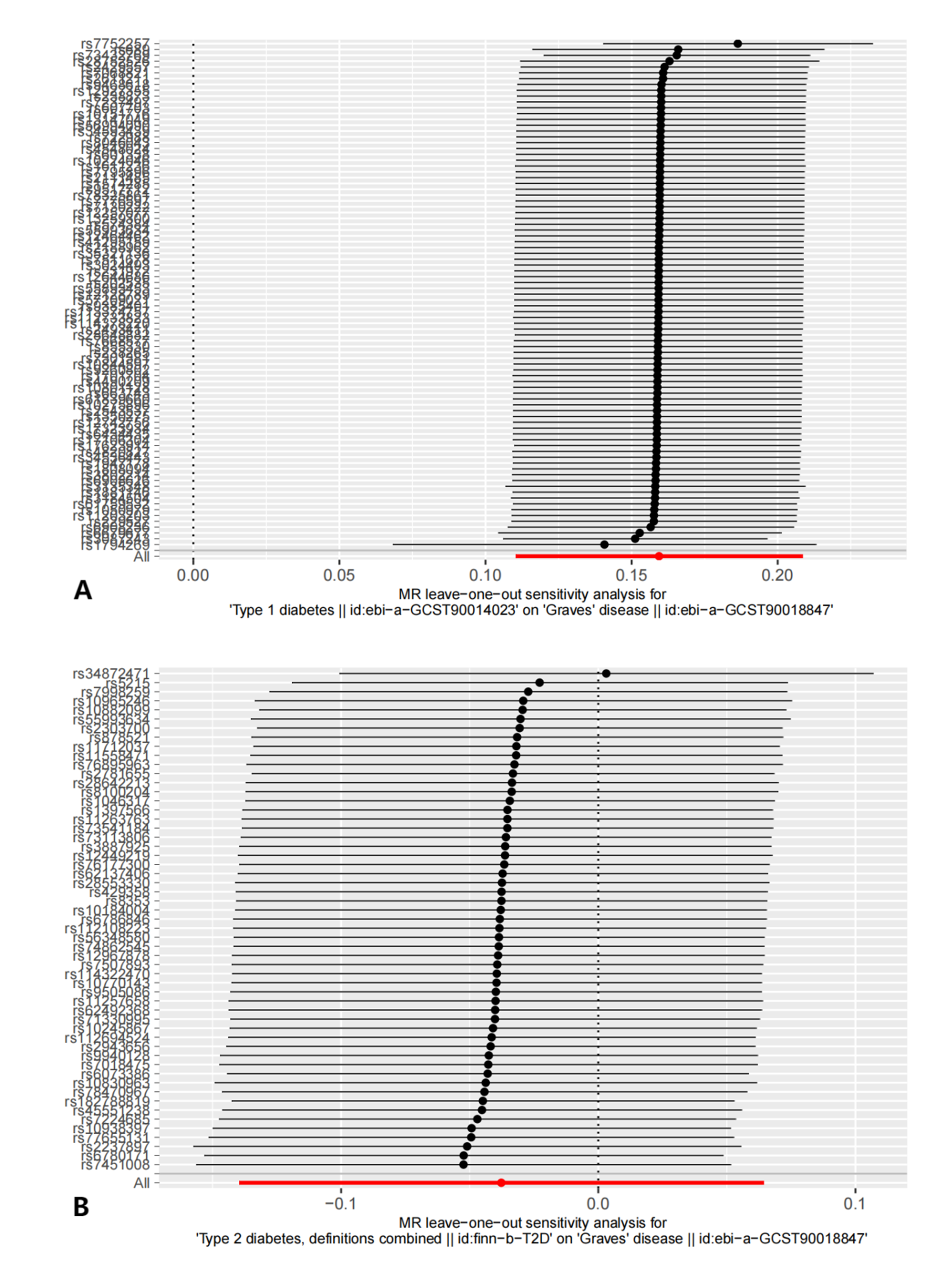 |  |  |
|  | **DISCUSSION** |  |  |  |
| 14 | **Key results** | GD and both T1D and T2D were causally linked in this study, indicating that a genetic predisposition to GD increases the risk of both T1D and T2D. However, reverse MR suggested that genetic susceptibility to T1D increases the risk of developing GD, while T2D does not. Furthermore, there is inadequate evidence to suggest that abnormal TSH, TPO, and Tg levels increase the risk of developing T1D or T2D in patients with GD. |  |  |
| 15 | **Limitations** | First, database limitations prevented the inclusion of indicators related to thyroid function, such as thyroid hormone, TSH receptor, anti-Tg, and anti-TPO. Additional research is required to examine the relationships between other indicators and DM. Second, our study did not investigate the causal relationship between Hashimoto's thyroiditis and T1D or T2D. Future studies should explore autoimmune thyroiditis. Third, the scope of this study was restricted to the European population. Therefore, additional research is necessary to verify whether our findings apply to other research groups. |  |  |
| 16 | **Interpretation** |  |  |  |
|  | a) | Meaning: A two-sample MR analysis was used to find evidence of bidirectional causality between GD and T1D and unidirectional causality between GD and T2D. No significant causal relationship between TSH, TPO, or Tg and T1D or T2D was found in the MVMR analysis. These findings provide new insights into the control of DM. However, due to the limitations of the database, the types of indicators reflecting thyroid function included in the study were not comprehensive. Consequently, in our subsequent studies, we will focus on the effects of thyroid-related hormones, antibodies, and biological enzymes on T1D and T2D |  |  |
|  | b) | Mechanism:   1. imbalance between Treg and Th17 cells   Some studies have reported varying degrees of functional impairment, decreased Treg cell efficiency, and enrichment of T helper 17 cells (Th17) in patients with GD. Th17 cells play a role in promoting inflammation and immune responses, whereas Tregs act as immunosuppressors. There may be a link between the onset and progression of DM (types 1 and 2) and an imbalance between Tregs and Th17 cells. A population-based study conducted in Finland revealed a significant correlation between T1D and other autoimmune diseases, including GD. The study found a significant association between hyperthyroidism and an increased risk of developing T1D (OR 2.98 [2.27–3.90]). In addition, GD has been identified as a primary cause of hyperthyroidism. In a separate study, it was found that out of 491 patients diagnosed with T1D, 122 tested positive for the TPO antibody, including 15 with autoimmune thyroid disease. In addition, a study of 500 individuals reported that patients with autoimmune thyroid disorders were more likely to develop other autoimmune diseases. Among them, researchers found T1D in 3.1% of patients with GD, confirming our finding that genetic susceptibility to GD increases the risk of developing T1D. The onset and progression of T2D is a complex process involving multiple mechanisms. Several studies have reported on the involvement of immune factors in the pathogenesis of this disease. Obesity-associated chronic inflammation is an important factor in the predisposition to T2D. Multiple studies have shown that an imbalance between Treg and Th17 cells, specifically a deficiency of Tregs and an excess of Th17 cells, is linked to obesity, insulin resistance, and T2D. Some studies have suggested that systemic Treg defects are associated with diabetic nephropathy, retinopathy, and diabetic foot ulcers. An increasing number of Tregs in the body has been shown to improve the severity of diabetes in some animal studies. Fengjie et al. discovered that Tregs can promote the mobilization of endothelial progenitor cells, anti-inflammatory effects, and cytoprotection. This can inhibit vascular endothelial hyperplasia and alleviate the vascular degeneration caused by elevated blood glucose levels in diabetic pigs. One study found that CD4+ Tregs decreased in the visceral tissues of patients with T2D. This suggests that an imbalance between the quality and capability of Treg and Th17 cells is involved in the development of GD, T1D, and T2D. This finding is consistent with the bidirectional causal relationship between GD and T1D. In contrast, the etiology of T2D is multifactorial, with immunological factors being one of the many contributors to its onset or progression. Consequently, patients with GD may have an increased risk of T2D due to the disruption of immune system homeostasis, while T2D does not necessarily increase the risk of GD.  (2) Chronic inflammation  Chronic inflammation should also be taken into consideration. It is common for autoimmune diseases to be accompanied by a chronic inflammatory response. Researchers have revealed that some cytokines, including transforming growth factor beta (TGF-β), interleukin-6 (IL-6), IL-4, IL-5, IL-10, IL-13, IL-1β, and interferon-gamma (IFN-γ), are associated with GD. T1D is associated with chronic inflammation 62, and several studies have reported that cytokines, such as IFN-γ, tumor necrosis factor (TNF)-α, and IL-1β, contribute to the pathogenesis of T1D. We speculate that chronic inflammation triggered by GD may contribute to the development of T1D, and vice versa. This finding suggests a bidirectional causal relationship between GD and T1D. In recent research, T2D has been increasingly associated with chronic inflammation. Chronic inflammation with increased levels of various cytokines contributes to the development of T2D. It has been reported that conventional type 1 dendritic cells can promote insulin resistance by increasing IFN-γ production. IL1-β, IL-6, IL-8, TNF-α, NF-κB, and MAPK were also significantly increased in the T2D and prediabetes groups compared to non-diabetic groups, indicating their role as pro-inflammatory factors. Chronic inflammation resulting from GD-induced increases in the levels of inflammatory factors, such as IFN-γ, IL-1β, and TNF-α in vivo, may contribute to insulin resistance and increase the risk of developing T2D by affecting insulin sensitivity. It is essential to note that this suggestion is based on objective evidence rather than subjective evaluation. Chronic inflammation is not the sole factor in the development of T2D. Therefore, the complex etiology of T2D indicates that GD development is not necessarily increased by T2D. |  |  |
|  | c) | Clinical relevance: Because GD increases the risk of both T1D and T2D diabetes, patients with GD should be more aware of early screening for diabetes than the general population. people with GD can also avoid both T1D and T2D or delay the onset of them by living a healthier lifestyle in the early period. |  |  |
| 17 | **Generalizability** | This study is based on a European population and we will include other populations in future studies to increase the generalizability of the results. |  |  |
|  | **OTHER INFORMATION** |  |  |  |
| 18 | **Funding** | none |  |  |
| 19 | **Data and data sharing** | Genetic data associated with GD, TPO, Tg, TSH, and T1D can be found in the IEU Open GWAS database (<https://gwas.mrcieu.ac.uk/>). The GWAS information for T2D was gathered from the FinnGen biobank database ([mailto:@online{finngen),](mailto:@online%7bfinngen,). |  |  |
| 20 | **Conflicts of Interest** | The authors declare that the research was conducted in the absence of any commercial or financial relationships that could be construed as a potential conflict of interest. |  |  |

This checklist is copyrighted by the Equator Network under the Creative Commons Attribution 3.0 Unported (CC BY 3.0) license.

1. Skrivankova VW, Richmond RC, Woolf BAR, Yarmolinsky J, Davies NM, Swanson SA, et al. Strengthening the Reporting of Observational Studies in Epidemiology using Mendelian Randomization (STROBE-MR) Statement. JAMA. 2021; under review.

2. Skrivankova VW, Richmond RC, Woolf BAR, Davies NM, Swanson SA, VanderWeele TJ, et al. Strengthening the Reporting of Observational Studies in Epidemiology using Mendelian Randomisation (STROBE-MR): Explanation and Elaboration. BMJ. 2021;375:n2233.
